# Nicotine Withdrawal Syndrome in Intensive Care Patients—Preventive and Therapeutic Implications

**DOI:** 10.3390/medsci14030374

**Published:** 2026-07-04

**Authors:** Renata Piotrkowska, Aneta Miszewska, Sandra Lange, Wioletta Mędrzycka-Dąbrowska, Sabina Krupa-Nurcek

**Affiliations:** 1 Department of Surgical Nursing, Faculty of Health Sciences, Medical University of Gdansk, 80-211 Gdańsk, Poland; rpiotrkowska@gumed.edu.pl; 2 Department of Anaesthesiology Nursing & Intensive Care, Faculty of Health Sciences, Medical University of Gdansk, Dębinki 7, 80-211 Gdańsk, Poland; aneta.miszewska@gumed.edu.pl; 3 Department of Internal and Pediatric Nursing, Medical University of Gdansk, Dębinki 7, 80-211 Gdańsk, Poland; 4 Department of Surgery, Faculty of Medicine, Collegium Medicum, University of Rzeszów, 35-310 Rzeszów, Poland

**Keywords:** nicotine dependence, nicotine withdrawal syndrome, delirium, intensive care unit

## Abstract

Introduction: Nicotine dependence is a chronic disorder with both psychological and somatic components which, in the intensive care unit (ICU) setting, may affect the course of treatment through mechanisms related both to long-term nicotine exposure and to the consequences of its abrupt cessation. The aim was to collect and map the current knowledge on smoking-related complications, the prevalence of nicotine withdrawal symptoms in this group, and to identify and describe interventions used to prevent or alleviate nicotine withdrawal symptoms in patients hospitalised in the ICU. Methods: The review included sources retrieved from databases such as PubMed, CINAHL, Scopus, Web of Science, and the Cochrane Library, published in English, that met the PCC criteria, with no time restrictions. Results: Forty-four sources were included. Twenty-nine contributed evidence on smoking-related status as an exposure or associated factor, five explicitly focused on abrupt nicotine cessation or nicotine withdrawal syndrome, and fifteen addressed interventions; categories overlapped. Delirium was the most frequently investigated outcome in smoking-related exposure studies. Withdrawal-focused sources reported or discussed nonspecific manifestations, including agitation, restlessness, anxiety, craving, and delirium-like presentations, but no validated ICU-specific diagnostic approach or robust prevalence estimate was identified. NRT was the only intervention evaluated. Conclusions: Smoking-related status was associated with agitation and delirium in several observational studies; however, heterogeneous exposure definitions and inconsistent evidence syntheses preclude causal or general prognostic conclusions. Evidence specific to nicotine withdrawal syndrome was limited, and the effectiveness and safety of NRT remain uncertain. Implications for clinical practice included routine identification of nicotine dependence at ICU admission, early monitoring of withdrawal symptoms, individualisation of sedation management, careful and selective consideration of nicotine replacement therapy (NRT), education of the therapeutic team, planning of further care, and smoking cessation interventions.

## 1. Introduction

Nicotine dependence is a chronic disorder, with both psychological and somatic components; it is characterised by compulsive and persistent regular nicotine intake. In medical terms, chronic smoking is referred to as tobacco or nicotine dependence, tobacco addiction, or nicotinism [1].

Nicotine dependence is manifested by a strong internal craving and impaired control over nicotine use. The need to use nicotine becomes prioritised over other activities and persists as a habitual behaviour despite awareness of the harm or negative consequences. The need to use nicotine results from biological dependence, which is often accompanied by a subjective craving for nicotine, particularly in specific social situations or emotional states. In dependent individuals, typical physiological manifestations are also observed, such as the development of tolerance to nicotine and the occurrence of withdrawal symptoms after its discontinuation or reduction [2].

Active smokers constitute a substantial proportion of patients admitted to intensive care units (ICUs), in whom specific clinical needs resulting from abrupt nicotine cessation are often insufficiently addressed in routine medical practice [3,4]. Previous studies indicate that nicotine dependence may affect the course of treatment in the ICU through mechanisms related both to chronic nicotine exposure and to the consequences of its abrupt cessation [3,4,5,6,7,8,9,10,11,12]. The literature lacks comprehensive reviews synthesising the current state of knowledge on nicotine dependence in the population of patients hospitalised in ICUs and on the available therapeutic management strategies. Therefore, there is a need for a broader review and synthesis of the available scientific evidence in this area.

### 1.1. Background

Among patients admitted to the intensive care unit (ICU), 25–47% are active smokers. The effects of nicotine in the body of a mechanically ventilated patient are similar to those observed in other individuals; however, in the ICU setting, they are of particular clinical importance because nicotine affects the nervous, cardiovascular, and respiratory systems, as well as the body’s inflammatory response. Nicotine activates the sympathetic nervous system by increasing catecholamine release. As a result, it causes an increased heart rate, elevated blood pressure, increased myocardial contractility, and vasoconstriction. Nicotine exerts its effects through nicotinic acetylcholine receptors (nAChRs) in the central and peripheral nervous systems. Their activation results in the release of multiple neurotransmitters, including dopamine, noradrenaline, serotonin, and acetylcholine [13]. Consequently, active smokers exhibit greater agitation, more frequent self-removal of devices, a greater need for physical restraint, and receive higher doses of sedatives, neuroleptics, and analgesics. Agitation may be a consequence of nicotine withdrawal, but it may also result from delirium or withdrawal from alcohol and/or illicit drugs. To date, it remains unclear whether smoking is associated with a higher risk of delirium during an ICU stay [8]. Nicotine withdrawal syndrome causes affective, cognitive, and somatic symptoms such as irritability, anxiety, restlessness, difficulty concentrating, insomnia, and increased appetite. Symptoms occur within 24 h of smoking cessation, peak after 3 days, and resolve approximately 4 weeks after cessation [14].

### 1.2. Aim

The aim of this scoping review was to collect and map the current knowledge on smoking-related complications, the prevalence of nicotine withdrawal symptoms in this group, and to identify and describe interventions used to prevent or alleviate nicotine withdrawal symptoms in patients hospitalised in the ICU.

## 2. Methods

### 2.1. Study Design

A scoping review method was selected. Scoping reviews draw on evidence from a variety of research methodologies and may also include evidence from non-research sources. This scoping review was conducted according to the methods described in the Joanna Briggs Institute Methodology Manual for Scoping Reviews [15] and using the Preferred Reporting Items for Systematic Reviews and Meta-analysis for Scoping Reviews (PRISMA-ScR) recommendations [16]. This scoping review was not registered in a public registry.

### 2.2. Review Questions

We developed research questions that clearly defined the PCC elements of this scoping review as follows:

1. What are the possible complications in smokers hospitalised in the ICU (with nicotine dependence as a risk factor)?
2. What is known about the frequency and clinical presentation of nicotine withdrawal syndrome or withdrawal-related symptoms in adult ICU patients?
3. What interventions are used to prevent nicotine withdrawal symptoms in intensive care patients?

### 2.3. Identifying Relevant Studies

Two authors (R.P. A.M.) systematically searched the following databases: PubMed, CINAHL, Scopus, Web of Science, and Cochrane Library. Five databases were used to access a wide range of English-language literature, without time limit. PCC, inclusion, and exclusion criteria are presented in Table 1. In addition, the researchers checked the reference list of existing literature reviews to identify eligible records that may have been missed during the search. A formal grey literature search was not conducted. This source was interpreted cautiously because it had not been published as a peer-reviewed journal article. The final search was conducted in February 2026. Search strategies are available in the Supplementary Materials.

#### 2.3.1. Population

Studies conducted in adult ICU patients with documented nicotine dependence (active smokers, nicotine-dependent individuals, and individuals with a history of smoking), regardless of the reason for admission, were included in this scoping review. Adults were defined as individuals aged 18 years or older.

#### 2.3.2. Concept

Risk factors for complications in patients with nicotine dependence in the ICU (e.g., infections, agitation, delirium, prolonged mechanical ventilation, and mortality); the prevalence and characteristics of nicotine withdrawal syndrome in the ICU; and interventions to prevent withdrawal syndrome or alleviate its symptoms (e.g., nicotine replacement therapy, medications, and non-pharmacological strategies).

#### 2.3.3. Context

Various types of intensive care units were considered. An intensive care unit was defined as a hospital ward that provides care to critically ill patients with life-threatening injuries and illnesses.

### 2.4. Study Selection

Study selection was conducted in several stages. First, all identified records were imported into a reference management system (EndNote), and then duplicates were removed. Subsequently, two independent reviewers conducted an initial screening based on titles and abstracts, applying predefined inclusion and exclusion criteria based on the PCC framework. Publications deemed potentially relevant were then evaluated in full text for eligibility. The final decision on study inclusion was made after analysing the full texts. In cases of disagreement, decisions were resolved through discussion and consensus, with the involvement of a third reviewer if necessary. The same PCC-based eligibility criteria were applied to all full-text sources, regardless of the publication format. The doctoral dissertation included in the review was one of the reports identified during database searching. Because it reported original empirical ICU data, was available in full text, and met the predefined PCC-based eligibility criteria, it was retained in the final evidence map.

### 2.5. Charting the Data

Data extraction was undertaken independently by two reviewers. The information charted from each included source comprised the first author’s name, year of publication, country, study design, setting, sample size, main findings, evidence-focus category, and, where applicable, intervention type and intervention category. The authors performed the extraction using Microsoft Excel. The data extraction template was not pilot-tested; however, all reviewers used a shared coding manual to ensure reliability.

### 2.6. Collating, Summarising, and Reporting the Results

Data were collated and synthesised descriptively using a structured classification framework aligned with the three review questions [17]. For reporting purposes, the review question domains were expressed as descriptive evidence-focused categories: smoking-related exposure (SRE; RQ1), withdrawal-focused evidence (WFE; RQ2), and intervention-focused evidence (IFE; RQ3). Each source was assigned to one or more categories according to the review question (s) it addressed. SRE comprised sources in which smoking history, current or former smoking, or nicotine dependence—including definitions based on diagnostic codes—was analysed as an exposure or associated factor. WFE comprised sources that explicitly investigated abrupt nicotine cessation or nicotine withdrawal syndrome, assessed symptom domains framed by the source authors as potentially withdrawal-related, or addressed the clinical recognition of nicotine withdrawal. IFE comprised sources that evaluated or described an intervention intended to prevent or alleviate nicotine withdrawal. Multiple categories were permitted when a source addressed more than one review question. Sources evaluating nicotine replacement therapy (NRT) were not classified as WFE solely because NRT was administered or studied.

For sources classified as IFE, intervention characteristics were charted separately, including the intervention type, formulation, and broad category. These categories were developed iteratively from the charted data and refined during synthesis to support consistent classification across sources [17,18]. Intervention data were charted from eligible sources identified through the overarching PCC-based search strategy; no separate search was conducted exclusively for intervention studies. The resulting evidence map is presented in Table 2 and summarised narratively by review questions [3,5,6,7,8,9,10,11,12,13,19,20,21,22,23,24,25,26,27,28,29,30,31,32,33,34,35,36,37,38,39,40,41,42,43,44,45,46,47,48,49,50,51,52,53].

## 3. Results

### 3.1. Search Outcomes

**Figure 1:**
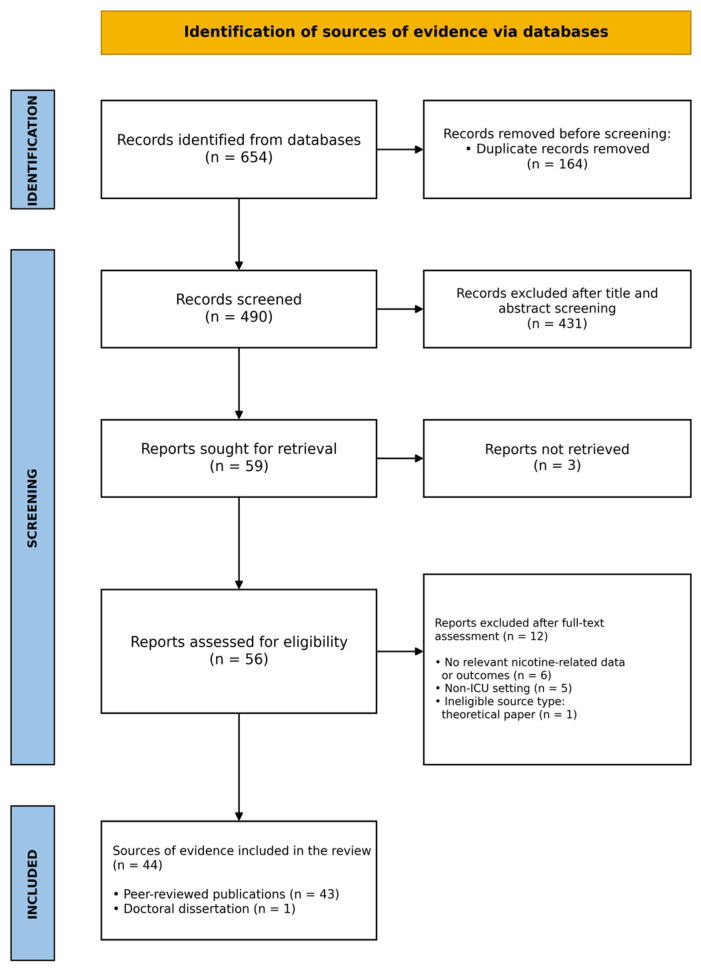
PRISMA-ScR flow diagram of source selection.

A total of 654 records were identified through database searching. Records retrieved from the searches were imported into EndNote. Duplicates were removed automatically based on DOI, followed by a manual verification of potential duplicates, including differences in title formatting, epub-ahead-of-print versus final versions, and records without a DOI. After deduplication, 164 duplicates were removed, leaving 490 records for title and abstract screening. During title and abstract screening, 431 records were excluded, and 59 reports progressed to full-text retrieval (i.e., records marked as include or maybe at this stage). The most frequent reasons recorded at the title/abstract stage were irrelevant concept (n = 237), conference abstract (n = 71), irrelevant concept + wrong population + wrong context (n = 58), wrong population (n = 19), irrelevant concept + wrong population (n = 19), and wrong population + wrong context (n = 14). Less frequent reasons included commentary (n = 6), wrong context (n = 4), letter to the editor (n = 1), and other/wording variants of “irrelevant concept” (n = 2). Of the 59 reports sought for retrieval, three could not be retrieved (full text unavailable) despite contacting the corresponding authors; full texts were not obtained. Therefore, 56 full-text reports were assessed for eligibility. Following the full-text assessment, 12 reports were excluded for the following reasons: wrong concept/no relevant nicotine outcomes (n = 6), wrong context (not ICU) (n = 5), and wrong study type (a theoretical paper) (n = 1). The included sources comprised 43 peer-reviewed publications and one doctoral dissertation identified during database searching. Figure 1 presents the PRISMA-ScR flow diagram, including the number of reports not retrieved and the reasons for full-text exclusion.

### 3.2. Characteristics of the Included Studies

Publication years and geographic distribution.

The 44 included sources were published between 1997 and 2025, with a median publication year of 2017. The distribution of publications over time suggested increasing attention to nicotine dependence, nicotine withdrawal, and related ICU outcomes in recent years. Four sources were published between 1997 and 2005, four between 2006 and 2010, ten between 2011 and 2015, eleven between 2016 and 2020, and fifteen between 2021 and 2025. The largest number of sources originated from the USA (n = 12) and China (n = 8), with the remaining sources distributed across Europe, North America, Asia, Australia, and the Middle East (Table 2) [3,5,6,7,8,9,10,11,12,13,19,20,21,22,23,24,25,26,27,28,29,30,31,32,33,34,35,36,37,38,39,40,41,42,43,44,45,46,47,48,49,50,51,52,53].

### 3.3. Study Design

The evidence base was methodologically heterogeneous. The most frequent designs were prospective observational cohort studies (n = 10), retrospective cohort or retrospective observational cohort studies (n = 8), systematic reviews/meta-analyses (n = 6), retrospective case–control studies (n = 5), and randomised controlled trials (n = 5). Other observational designs accounted for six sources. The remaining sources comprised narrative reviews (n = 2), one case report (n = 1), and one doctoral dissertation reporting original prospective observational pilot data from an ICU population (n = 1) (Table 2).

### 3.4. ICU Setting (General vs. Cardiac vs. Neuro)

Based on the “setting” field recorded for each included study, the ICU contexts were distributed as follows: general ICU (including general/mixed adult ICU settings), n = 24; cardiac ICU/cardiac surgery ICU settings, n = 5; neuro-ICU/neurosurgical ICU settings, n = 4; medical/surgical ICU and postoperative ICU settings, n = 10; and emergency ICU, n = 1.

Where reviews covered multiple ICU types or the ICU subtype was unclear, the setting was retained as reported (Table 2).

### 3.5. Evidence-Focus Classification and Mapping to the Review Questions (PCC-Aligned)

To distinguish associations with smoking-related status from evidence specifically concerning abrupt nicotine cessation, each included source was classified according to its evidence focus: smoking-related exposure (SRE), withdrawal-focused evidence (WFE), or intervention-focused evidence (IFE). SRE denoted sources in which smoking history, current or former smoking, or coded nicotine dependence was analysed as an exposure or associated factor. WFE denoted sources that directly assessed abrupt nicotine cessation or candidate withdrawal-related symptoms, or explicitly addressed the clinical recognition of nicotine withdrawal syndrome. IFE denoted sources in which an intervention intended to prevent or alleviate nicotine withdrawal was evaluated or described. More than one code could be assigned to a source. NRT-focused sources were not classified as WFE solely because NRT was administered or evaluated to prevent withdrawal. Classification as WFE reflects the focus of the source and does not indicate that nicotine withdrawal syndrome was confirmed using a validated ICU-specific diagnostic instrument or that nonspecific ICU manifestations were causally attributable to withdrawal.

The evidence-focus categories mapped to the review questions are as follows (Table 2): SRE/RQ1, n = 29; WFE/RQ2, n = 5; and IFE/RQ3, n = 15. These totals are not mutually exclusive. Of the 44 included sources, 28 were classified as SRE only [11,12,19,20,22,23,24,25,26,27,28,29,30,31,32,33,35,37,38,39,40,41,42,43,44,45,47,49], 1 as both SRE and WFE [5], 4 as both WFE and IFE [48,50,51,52], and 11 as IFE only [3,6,7,8,9,10,13,21,34,36,53].

### 3.6. Results by Review Question

RQ1—What complications are reported among smokers admitted to the ICU (smoking as a risk factor)?

Twenty-nine sources contributed to RQ1. Twenty-eight evaluated smoking history, current or former smoking, or coded nicotine dependence as an exposure or associated factor without directly assessing abrupt nicotine cessation or nicotine withdrawal syndrome [11,12,19,20,22,23,24,25,26,27,28,29,30,31,32,33,35,37,38,39,40,41,42,43,44,45,47,49]. Lucidarme et al. [5] explicitly investigated sudden nicotine abstinence and was therefore additionally classified as withdrawal-focused evidence. Accordingly, except for the explicitly identified withdrawal-focused source [5], the findings in this subsection represent associations with smoking-related status and should not be interpreted as effects of nicotine withdrawal syndrome.

Delirium was the most frequently examined outcome. Individual studies reported associations between smoking-related status and delirium or postoperative delirium, although the definitions of smoking exposure, methods of delirium assessment, and approaches to confounding adjustment varied [11,12,20,22,23,24,25,26,28,29,30,31,39,40,41,42,43,44,45]. For example, regular smoking was independently associated with delirium in mechanically ventilated patients (RR 2.366; 95% CI 1.277–4.382) [24], smoking history was independently associated with postoperative delirium after elective craniotomy (aOR 2.582; 95% CI 1.611–4.140) [25], and smoking was independently associated with delirium in a large sepsis cohort (OR approximately 1.44; 95% CI 1.28–1.61) [30]. After propensity score matching, coded nicotine dependence was associated with a modestly higher incidence of delirium (30.8% vs. 27.2%; *p* = 0.004), whereas mortality and length of stay did not differ significantly [28].

Findings from evidence syntheses were inconsistent. One meta-analysis reported an association between smoking and delirium (OR 1.55; 95% CI 1.11–2.00; I^2^ = 74.1%) [33]. In contrast, another meta-analysis found no statistically significant association in either univariate analyses (OR 1.01; 95% CI 0.81–1.25) or adjusted analyses (OR 1.61; 95% CI 0.83–3.10) [37], while a further review considered the available evidence inconclusive [35].

Evidence concerning agitation arose from two different evidence domains. de Almeida et al. evaluated smoking status as a risk factor and found that smoking was independently associated with agitation during the first seven ICU days (OR 4.49; 95% CI 1.33–15.17) [19]. Abrupt nicotine cessation and nicotine withdrawal syndrome were not assessed in that study. In contrast, Lucidarme et al. explicitly examined sudden nicotine abstinence. Agitation occurred more frequently in smokers than in non-smokers (64% vs. 32%; *p* = 0.0005), and active smoking remained independently associated with agitation in multivariable analysis (OR 3.13; 95% CI 1.45–6.74) [5]. Smokers also had more self-removal of tubes or catheters and required more supplemental sedatives, analgesics, neuroleptics, and physical restraints [5]. However, the study assessed nicotine dependence using the Fagerström Test and measured agitation and delirium using the Sedation–Agitation Scale and the Intensive Care Delirium Screening Checklist, respectively; it did not confirm nicotine withdrawal syndrome using a withdrawal-specific ICU diagnostic instrument. These findings therefore support an association in a withdrawal-focused context but do not establish that each agitation episode was caused by nicotine withdrawal.

Other outcomes examined directly in relation to smoking-related status included unplanned extubation or self-extubation [27,49], prolonged mechanical ventilation [47], ventilator-associated pneumonia in a prone-position subgroup [38], and pressure ulcers in one oncology ICU cohort [44]. Smoking history was independently associated with prolonged mechanical ventilation in the study by Cai et al. (OR 7.417; 95% CI 2.425–22.684) [47]. Smoking history also predicted unplanned extubation in one study [27]. In Atkins et al. [49], current smoking was more frequent among self-extubation cases, whereas restlessness/agitation and hospital-acquired adverse events, rather than smoking status, were reported as independent factors for self-extubation. Restlessness in that study was therefore not interpreted as evidence of nicotine withdrawal syndrome.

In several studies, longer mechanical ventilation times, longer ICU or hospital stays, device removal, reintubation, pressure ulcers, or mortality were reported primarily as outcomes of delirium or self-extubation rather than as outcomes independently attributable to smoking-related status [22,23,26,41,42,49]. These downstream outcomes were therefore not interpreted as direct effects of smoking or nicotine withdrawal. In Kotfis et al. [32], smokers were more common among patients extubated within 12 h; because smoking was not established as an independent determinant of earlier extubation, this finding was not interpreted as evidence of a protective effect.

As this was a scoping review, the findings were synthesised to map evidence domains rather than to estimate a pooled effect.

RQ2—What is known about the frequency and clinical presentation of nicotine withdrawal syndrome or withdrawal-related symptoms in adult ICU patients?

Five sources were classified as withdrawal-focused evidence [5,48,50,51,52]. Three provided primary empirical ICU data [5,48,52], and two were narrative reviews [50,51]. Kowalski et al. [3] and Cartin-Ceba et al. [21] were classified as intervention-focused rather than withdrawal-focused because their analyses were based on NRT exposure and nonspecific clinical outcomes and did not directly assess the occurrence, diagnostic criteria, or clinical phenotype of nicotine withdrawal syndrome.

The empirical evidence did not permit calculations of a pooled prevalence of nicotine withdrawal syndrome. The primary sources did not apply a common case definition or a validated ICU-specific diagnostic instrument and did not provide comparable denominator-based prevalence data. Lucidarme et al. evaluated the impact of sudden nicotine abstinence by comparing smokers with non-smokers and reported more agitation among smokers, but did not diagnose nicotine withdrawal syndrome using a withdrawal-specific instrument [5]. Carle’s prospective pilot study enrolled eight patients and assessed candidate withdrawal-related symptom domains, including anxiety, agitation, cigarette craving, and delirium-related measures; its findings were exploratory because of the very small sample, and only one participant received NRT [48]. Mayer et al. described five heavy smokers with brain injury who developed presumed nicotine withdrawal delirium after smoking cessation and improved following administration of a 21 mg transdermal nicotine patch; the authors did not establish causality [52].

The narrative reviews discussed agitation or restlessness, anxiety, craving, sleep disturbance, and delirium-like presentations as possible manifestations of nicotine withdrawal in critically ill patients [50,51]. These descriptions were not treated as confirmed cases of nicotine withdrawal syndrome or as prevalence data. The manifestations are nonspecific and overlap with delirium, pain, hypoxaemia, anxiety, mechanical ventilation-related distress, effects of sedative or opioid medications, and withdrawal from alcohol or other substances.

No included source applied a validated ICU-specific diagnostic instrument for nicotine withdrawal syndrome or provided a generalisable prevalence estimate. Therefore, the prevalence of nicotine withdrawal syndrome in ICU patients remains undetermined, and agitation, restlessness, or delirium should not be attributed to nicotine withdrawal solely on the basis of smoking history or nicotine dependence.

RQ3—What interventions are used to prevent or mitigate nicotine withdrawal in the ICU?

Fifteen sources were classified as intervention-focused evidence [3,6,7,8,9,10,13,21,34,36,48,50,51,52,53]. All concerned nicotine replacement therapy. These sources were used to map the interventions evaluated or described and their reported clinical outcomes. Agitation, delirium, restraint use, mortality, or other outcomes reported in comparisons of NRT with no NRT were not treated as evidence that nicotine withdrawal syndrome was present unless withdrawal symptoms were directly assessed. Transdermal nicotine patches were the most frequently reported formulation [3,6,8,9,10,13,21,34,50,51,52,53], whereas the NRT formulation was not specified in three sources [7,36,48]. No eligible source evaluated bupropion, varenicline, cytisine, or another pharmacological agent specifically for nicotine withdrawal in ICU patients, and no structured non-pharmacological protocol designed to mitigate nicotine withdrawal in the ICU was identified.

Evidence regarding NRT effectiveness and safety was inconsistent. In three small RCTs, de Jong et al. reported more time alive without delirium, sedation, or coma, with comparable serious adverse events and 30- and 90-day mortalities [8]. Pathak et al. observed a numerically shorter ICU stay (4.5 vs. 7.0 days) and fewer ventilator days (1.9 vs. 3.5), but the differences were not statistically significant [9]. Kanova et al. found no significant effect on delirium, ICU or hospital stay, mechanical ventilation, sedation, analgesia, or vasopressor use [10]. None of these trials assessed confirmed nicotine withdrawal syndrome using a validated ICU-specific instrument.

Observational findings were similarly inconsistent. Kerr et al. reported greater antipsychotic use (34.1% vs. 11.1%) and physical restraint use (29.4% vs. 9.5%) among patients receiving NRT [6]. Lee et al. reported higher hospital mortality with NRT (20% vs. 7%; adjusted OR 24.6; 95% CI 3.6–167.6) [7], whereas Cartin-Ceba et al. found no significant association with mortality after adjustment (OR 1.4; 95% CI 0.5–3.9) [21]. In patients with subarachnoid haemorrhage, NRT was associated with lower 3-month mortality (OR 0.12; 95% CI 0.04–0.37), but delirium (19% vs. 9%) and seizures (9% vs. 2%) were more frequent in the NRT group [13]. A meta-analysis of observational studies found higher odds of delirium with NRT (OR 4.03; 95% CI 2.64–6.15), but no significant difference in ICU mortality [34]. Conversely, a meta-analysis of three RCTs (n = 139) reported a shorter ICU stay (MD −3.06 days; 95% CI −5.88 to −0.25), without significant effects on mechanical ventilation, delirium, or vasopressor use; certainty of evidence was low or very low [36]. Overall, the available evidence did not demonstrate a consistent clinical benefit of NRT, and safety-related findings remained heterogeneous.

## 4. Discussion

Analysis of the 44 publications included in this review indicates substantial heterogeneity in terms of study design. Most of the available evidence derives from observational studies, which account for more than half of all analysed publications. Randomised controlled trials, which provide the highest level of evidence, constituted only a small proportion of the analysed studies and often included small samples, thereby limiting their statistical power and the generalisability of their findings. Moreover, studies evaluating nicotine replacement therapy showed inconsistent results, both with regard to its effect on delirium and on other clinical outcomes. It should be emphasised that the purpose of this scoping review was not to confirm the effectiveness of any specific intervention, but to map how nicotine dependence, withdrawal-related symptoms, and withdrawal-directed management have been studied in ICU populations. This distinction is important because the available evidence includes different source types, including one doctoral dissertation with pilot observational data.

### 4.1. Smoking-Related Exposure and ICU Outcomes

The largest evidence domain comprised sources in which smoking history, current or former smoking, or coded nicotine dependence was evaluated as an exposure or associated factor rather than as evidence of acute nicotine withdrawal. Delirium was the most frequently investigated outcome. Several observational studies reported adjusted associations between smoking-related status and delirium, including an RR of 2.366 (95% CI 1.277–4.382) in mechanically ventilated patients [24], an aOR of 2.582 (95% CI 1.611–4.140) for postoperative delirium after elective craniotomy [25], and an OR of approximately 1.44 (95% CI 1.28–1.61) in patients with sepsis [30]. In a large propensity-score-matched cohort, coded nicotine dependence was associated with a modest absolute increase in delirium incidence (30.8% vs. 27.2%; *p* = 0.004), but not with mortality or length of stay [28].

The overall evidence was not consistent. One meta-analysis reported an association between smoking and delirium (OR 1.55; 95% CI 1.11–2.00), with substantial heterogeneity (I^2^ = 74.1%) [33], whereas another found no statistically significant adjusted association (OR 1.61; 95% CI 0.83–3.10) [37]. A further systematic review considered the evidence inconclusive [35]. In addition, one matched case–control study identified former, but not active, smoking as an independent factor associated with delirium [39]. These differences should be interpreted in the context of heterogeneous exposure definitions, including current smoking, former smoking, any smoking history, and diagnosis-code-defined nicotine dependence, with consideration given to the variations in delirium assessment methods, differences between ICU populations, and inconsistent adjustment for potential confounders. Consequently, the available evidence supports study-specific associations with smoking-related status but does not establish that nicotine dependence causes delirium.

The evidence concerning agitation also arose from two distinct exposure frameworks. de Almeida et al. evaluated smoking status as a potential risk factor and reported an independent association with agitation during the first seven ICU days (OR 4.49; 95% CI 1.33–15.17) [19]. That study did not assess abrupt nicotine cessation or diagnose nicotine withdrawal syndrome. By contrast, Lucidarme et al. explicitly investigated sudden nicotine abstinence in mechanically ventilated patients. Agitation occurred in 64% of smokers and 32% of non-smokers (*p* = 0.0005), and active smoking remained independently associated with agitation after multivariable adjustment (OR 3.13; 95% CI 1.45–6.74) [5]. Smokers also required more supplemental sedatives, analgesics, neuroleptics, and physical restraints and had more frequent self-removal of tubes or catheters [5]. However, agitation and delirium were assessed using general ICU instruments rather than a withdrawal-specific diagnostic method. The findings therefore support an association in the context of abrupt nicotine cessation but do not demonstrate that each agitation episode represented nicotine withdrawal syndrome.

Evidence concerning broader clinical outcomes was more limited and did not support a uniform adverse prognostic effect. In one postoperative cardiac surgery cohort, smoking history was independently associated with prolonged mechanical ventilation (OR 7.417; 95% CI 2.425–22.684), although the wide confidence interval indicates considerable imprecision [47]. Smoking history was also associated with unplanned extubation in one study (OR approximately 3.2) [27]. In another study, current smoking was more frequent among patients who self-extubated, but restlessness or agitation and hospital-acquired adverse events—not smoking status—were reported as independent factors for self-extubation [49]. Conversely, the large propensity-score-matched analysis found no significant differences in mortality or length of stay between patients with and without coded nicotine dependence [28].

Several other sources reported prolonged ventilation, longer ICU or hospital stays, device removal, reintubation, complications, or mortality primarily as consequences of delirium or self-extubation rather than as outcomes independently attributable to smoking-related status [22,23,26,41,42,49]. Similarly, the greater proportion of smokers among patients extubated within 12 h in the study by Kotfis et al. was a baseline association and should not be interpreted as evidence of a protective effect of smoking [32]. Overall, the mapped evidence does not support a general causal conclusion that smoking history or nicotine dependence independently worsens overall ICU prognosis.

### 4.2. Nicotine Withdrawal Syndrome in the ICU: Frequency and Clinical Presentation

Only five sources explicitly focused on abrupt nicotine cessation, nicotine withdrawal syndrome, or symptom domains interpreted by the source authors as potentially withdrawal-related [5,48,50,51,52]. Of these, three provided primary empirical ICU data [5,48,52], whereas two were narrative reviews [50,51]. This evidence base was substantially smaller and methodologically weaker than the literature evaluating smoking-related status as an exposure.

The available empirical evidence did not permit estimation of the prevalence of nicotine withdrawal syndrome in ICU patients. Lucidarme et al. examined sudden nicotine abstinence but did not diagnose the syndrome using a withdrawal-specific instrument [5]. Carle’s prospective pilot study included only eight participants and explored anxiety, agitation, cigarette craving, and delirium-related measures; its findings were therefore exploratory [48]. Mayer et al. described five heavy smokers with brain injury who developed presumed nicotine-withdrawal-related agitated delirium 2–10 days after smoking cessation and improved after receiving a 21 mg transdermal nicotine patch [52]. However, the authors explicitly acknowledged that causality could not be established and that the observed improvement might have reflected the underlying neurological condition, the natural clinical course, or other concurrent factors.

Across the withdrawal-focused sources, agitation or restlessness, anxiety, craving, sleep disturbance, increased requirements for sedative or behavioural management, and delirium-like presentations were reported or discussed as possible manifestations [5,48,50,51,52]. None of these manifestations is specific to nicotine withdrawal. They overlap substantially with delirium, pain, hypoxaemia, anxiety, mechanical ventilation-related distress, sleep disruption, medication effects, and withdrawal from alcohol, opioids, benzodiazepines, or other substances. Narrative descriptions of such symptoms should therefore not be treated as confirmed cases or as prevalence data.

No included source applied a validated ICU-specific diagnostic instrument or a common case definition for nicotine withdrawal syndrome. Furthermore, the primary studies did not provide comparable denominator-based data that would support pooled prevalence estimation. The appropriate clinical implication is therefore to consider abrupt nicotine cessation as one possible contributor within a broader differential assessment rather than to attribute agitation, restlessness, or delirium to nicotine withdrawal solely on the basis of smoking history or nicotine dependence.

Future research should use prospective designs with standardised assessment of smoking status and degree of dependence, clearly documented timing of smoking cessation, serial measurement of candidate withdrawal symptoms, and systematic evaluation of competing causes. Development and validation of an ICU-specific diagnostic approach would be necessary before the frequency, clinical phenotype, and prognostic significance of nicotine withdrawal syndrome can be established. This diagnostic uncertainty must also be considered when interpreting intervention studies because changes in nonspecific outcomes, such as agitation or delirium, do not necessarily demonstrate successful treatment of confirmed nicotine withdrawal.

### 4.3. Interventions Used to Prevent or Mitigate Nicotine Withdrawal in the ICU

The intervention-focused evidence should be interpreted separately from the five withdrawal-focused sources [5,48,50,51,52]. Fifteen sources were classified as intervention-focused [3,6,7,8,9,10,13,21,34,36,48,50,51,52,53], and all addressed nicotine replacement therapy. Importantly, NRT-focused sources were not considered evidence that nicotine withdrawal syndrome was present solely because NRT was administered or evaluated. Unless withdrawal symptoms were directly assessed, outcomes such as agitation, delirium, restraint use, sedative requirements, mortality, or length of stay were interpreted as clinical outcomes associated with the intervention rather than as confirmation of nicotine withdrawal syndrome.

Across these intervention-focused sources, findings regarding the effectiveness and safety of NRT were inconsistent. A systematic review published in 2016 analysed the use of NRT in active smokers hospitalised in the ICU. The results were inconsistent: some studies indicated a reduction in withdrawal symptoms, others linked NRT with increased agitation, and some showed no clear benefit [3]. Similarly, a meta-analysis published in 2017, which included various studies, found that NRT use was associated with an increased incidence of delirium in ICU patients, although it did not significantly affect mortality or ICU length of stay [34]. The prospective observational study by Cartin-Ceba et al. [21] suggests a possible neutral safety profile of NRT, but does not provide clear confirmation of its effectiveness in alleviating nicotine withdrawal symptoms. Other studies suggested that NRT was associated with greater use of antipsychotic medications and a higher need for physical restraints [6], while still others concluded that the evidence is insufficient to formulate clear recommendations regarding the use of NRT in the ICU [50,51,52,53].

There is a clear need for high-quality studies using appropriate tools to assess nicotine withdrawal symptoms in order to determine the effectiveness and safety of NRT and other methods unequivocally. Although medications such as bupropion, varenicline, and cytisine are used in the general population for the treatment of tobacco dependence, clinical data on their use in the ICU are lacking, which precludes recommendations for this patient group. Non-pharmacological interventions also play an important role in the treatment of nicotine dependence. The World Health Organization’s recommendations emphasise the importance of brief medical interventions and psychological support as core elements of tobacco dependence treatment [2]. Effective strategies include individual and group counselling, interventions based on the principles of motivational interviewing, and techniques derived from cognitive behavioural therapy, which help patients modify smoking-related behavioural patterns and develop skills to cope with stress and nicotine cravings [52].

At the same time, it should be emphasised that most of the available studies evaluating the effectiveness of non-pharmacological methods for nicotine dependence treatment have been conducted in ambulatory populations, involving individuals who are consciously attempting to stop smoking. Data on the use of these methods in hospitalised patients, particularly in intensive care units, remain very limited. Critically ill patients are often unable to participate actively in conventional forms of behavioural therapy because of a severe clinical condition, sedation, or the need for mechanical ventilation. Consequently, evidence is still lacking on whether non-pharmacological interventions may be effective in preventing or alleviating symptoms of nicotine withdrawal syndrome in this specific group of patients. Addressing this research gap is important from the perspective of optimising the care of critically ill patients and developing safe and effective management strategies for abrupt interruption of nicotine exposure during hospitalisation [54,55].

## 5. Strengths and Limitations

This review applied a broad search strategy across multiple databases, increasing the likelihood of identifying the full range of available evidence. Attempts were also made to contact corresponding authors when full-text access was unavailable, which supported maximising data completeness. The inclusion of a relatively large number of studies (n = 44), together with the absence of time restrictions, allowed for a comprehensive overview of the topic under investigation.

A limitation of the review was the absence of a developed and registered protocol, which may affect methodological transparency. The search strategy was mainly based on databases. At the same time, to minimise the risk of omitting relevant studies, the reference lists of other manuscripts were additionally analysed to identify potentially relevant sources. A formal grey literature search was not conducted. Therefore, potentially relevant conference proceedings, institutional reports, and academic works may have been missed.

The search strategy did not incorporate controlled vocabulary (e.g., MeSH terms or MH descriptors in CINAHL), which may have reduced search sensitivity. Only English-language publications were included, which may have resulted in the omission of studies published in other languages. In addition, the identification of interventions for the prevention and treatment of nicotine withdrawal symptoms constituted a secondary analysis and was not a distinct objective of this review, which may have limited the completeness of the data obtained. Moreover, the findings in this area were inconclusive. Therefore, it appears justified to conduct a separate, focused systematic review dedicated exclusively to interventions used in ICU patients.

## 6. Implications for Clinical Practice

### 6.1. Routine Identification of Nicotine Dependence at ICU Admission

A smoking history and assessment of the degree of nicotine dependence should form part of the standard patient assessment at admission to the intensive care unit. Early identification of active smoking enables recognition of patients at increased risk of agitation and delirium.

### 6.2. Early Monitoring of Withdrawal Symptoms

In patients with a history of nicotine dependence, enhanced monitoring should be undertaken for psychomotor agitation, increased sedation requirements, and difficulties in managing mechanical ventilation. These symptoms may represent the first manifestations of withdrawal syndrome in the ICU setting.

### 6.3. Integration of Nicotine Dependence into Delirium Prevention Protocols

Nicotine dependence should be considered a potentially modifiable risk factor in delirium prevention strategies, including optimisation of sedation, pain control, improvement of sleep quality, and early mobilisation.

### 6.4. Individualisation of Sedation Management

Patients with nicotine dependence may require higher doses of sedative medications. A balance is needed between controlling agitation and avoiding excessive sedation, which itself increases the risk of delirium and prolonged mechanical ventilation.

### 6.5. Careful and Selective Consideration of Nicotine Replacement Therapy (NRT)

Given the inconsistent and heterogeneous evidence, NRT cannot be recommended routinely for all ICU smokers. If NRT is considered, the decision should be individualised, taking into account the patient’s smoking history and degree of nicotine dependence, as well as relevant comorbidities and the reason for ICU admission, severity of critical illness, NRT formulation and dose, and the timing of initiation relative to abrupt smoking cessation. These factors should be regarded as clinical considerations rather than established predictors of benefit or harm because the included studies did not consistently report or stratify outcomes according to them. No optimal NRT regimen or clearly defined subgroup of ICU patients has yet been established.

### 6.6. Education of the Therapeutic Team

Healthcare professionals should be aware that abrupt nicotine withdrawal may affect the clinical course of treatment in the ICU. Taking this factor into account in daily practice may contribute to earlier identification of patients at risk of neurocognitive complications.

### 6.7. Planning of Further Care and Smoking Cessation Interventions

ICU hospitalisation may represent an opportunity for smoking cessation intervention. Once the patient’s condition has stabilised, implementation of smoking cessation support programmes should be considered, as this may improve their long-term prognosis.

### 6.8. Proposed ICU Admission Protocol for Nicotine-Dependent Patients

To facilitate clinical application at ICU admission, we propose a structured approach focusing on two main pillars: 1. Information to Check (Baseline Evaluation) Smoking Status and History: Confirm current vs. former smoking status, duration of smoking, and daily tobacco consumption (calculate pack-years). Nicotine Dependence Severity: Evaluate the dependence level using validated tools (e.g., Fagerström Test for Nicotine Dependence) or by proxy (e.g., time to first cigarette after waking). Alternative Nicotine Sources: Check for recent use of electronic cigarettes, heated tobacco products, or nicotine replacement therapy (NRT) prior to admission. 2. Symptoms to Observe (Active Monitoring) Nicotine Withdrawal Symptoms: Monitor closely for hyperirritability, anxiety, restlessness, insomnia, unexplained tachycardia, or intense cravings. Delirium and Cognitive Changes: Routinely screen for cognitive fluctuations, inattention, or altered level of consciousness, paying specific attention to hyperactive or hypoactive features. Sedation and Agitation Levels: Continuously track agitation or sedation depth (e.g., Richmond Agitation-Sedation Scale [RASS]) to properly balance sedative titration and withdrawal management.

## 7. Conclusions

Smoking-related status was associated with agitation and delirium in several observational ICU studies, although the magnitude and consistency of these associations varied. Representative adjusted estimates included ORs of 3.13 and 4.49 for agitation and an RR of 2.366, an aOR of 2.582, and an OR of 1.44 for delirium. One cohort associated smoking history with prolonged mechanical ventilation (OR 7.417), whereas a large propensity-score-matched cohort found no significant differences in mortality or length of stay. Accordingly, the available evidence does not establish that smoking history or nicotine dependence independently worsens one’s overall ICU prognosis.

Evidence specifically addressing nicotine withdrawal syndrome was sparse and did not provide a robust prevalence estimate or a validated ICU-specific diagnostic approach. Nicotine replacement therapy was the only intervention evaluated, but findings concerning its effectiveness and safety were inconsistent and do not support routine use in all ICU smokers.

Further prospective studies using standardised definitions and assessment methods are required.

## Figures and Tables

**Table 1:** PCC, inclusion, and exclusion criteria.

	Inclusion Criteria	Exclusion Criteria
Population (P)	• Adult patients (≥18 years) • Hospitalised in the ICU • Patients with current or past nicotine dependence (active smokers, nicotine-dependent individuals, individuals with a history of tobacco smoking)	• Paediatric patients (<18 years) • Patients without data on smoking/nicotine status • Patients hospitalised in settings other than the ICU (e.g., medical, surgical, or postoperative wards)
Concept (C)	• Studies addressing one or more of the following issues: risk factors for complications in patients with nicotine dependence in the ICU; occurrence, symptoms, and consequences of nicotine withdrawal syndrome; pharmacological and non-pharmacological interventions to prevent/alleviate withdrawal syndrome	• Studies not related to nicotine dependence or nicotine withdrawal syndrome • Addictions other than nicotine dependence (e.g., alcohol, opioids, benzodiazepines)
Context (C)	• ICUs of various types	• Studies concerning other clinical contexts (e.g., long-term care, palliative care)
Study type (S)	• Reviews (various types) • Meta-analyses • Quantitative studies • Qualitative studies • Intervention studies (RCTs, non-RCTs) • Observational studies (prospective, retrospective) • Case reports • Doctoral dissertations reporting original empirical data, available in full text	• Letters to the editor • Editorials • Commentaries • Conference abstracts • Non-full-text sources • Master’s theses and other academic works not reporting original empirical ICU data
Years considered/time period	Without time limit	N/A
Language	English	Other languages

**Table 2:** Characteristics, evidence focus, and key findings of the included sources of evidence (n = 44).

First Author	Country	Study Design	Setting	Number of Participants	Main Findings	Evidence Focus (Review Question)	Intervention Code	Intervention Category
Kowalski M. et al., 2016 [3]	Australia	Systematic review	Adult current smokers admitted to ICU; NRT used to manage nicotine withdrawal; delirium/agitation assessed variably across studies	6 studies included (1033 patients)	This systematic review evaluated whether NRT reduced agitation or delirium in critically ill smokers. Findings were inconclusive: three included studies reported an association between NRT and increased agitation or delirium, one found neither benefit nor harm, and two reported improvement in symptoms attributed by the original authors to nicotine withdrawal. Outcome definitions and assessment methods were inconsistent, and the review did not directly estimate the occurrence or clinical phenotype of NWS.	IFE (RQ3)	NRT—transdermal nicotine patch	Pharmacological
Lucidarme O. et al., 2010 [5]	France	Prospective observational cohort study	Two adult ICUs; included patients requiring respiratory support and mechanical ventilation > 48 h; NRT was prohibited during the study	144 total; 44 smokers, 100 non-smokers	Agitation occurred more frequently in smokers than in non-smokers (64% vs. 32%; *p* = 0.0005), whereas delirium incidence did not differ significantly. Smokers had more self-removal of tubes/catheters and required more supplemental sedatives/analgesics, neuroleptics, and physical restraints. Active smoking was independently associated with agitation (OR 3.13; 95% CI 1.45–6.74). The study explicitly examined sudden nicotine abstinence; however, NWS was not diagnosed using a withdrawal-specific ICU instrument.	SRE (RQ1); WFE (RQ2)		
Kerr A. et al., 2016 [6]	Australia	Retrospective case–control study	31-bed mixed medical-surgical ICU; active smokers receiving transdermal NRT matched to active smokers without NRT (age/sex/APACHE II)	252 (126 NRT, 126 control)	The NRT group had higher antipsychotic use (34.1% vs. 11.1%), more frequent use of physical restraints (29.4% vs. 9.5%), and longer intubation duration; 30-day mortality did not differ significantly.	IFE (RQ3)	NRT—transdermal nicotine patch	Pharmacological
Lee AH. et al., 2007 [7]	USA	Retrospective case–control study	Medical ICU, tertiary academic hospital	180 (90 NRT + 90 controls)	Among active smokers admitted to the ICU, exposure to NRT was associated with higher hospital mortality (20% vs. 7%). After adjustment for illness severity (APACHE III-predicted mortality) and invasive mechanical ventilation, NRT remained independently associated with increased mortality (OR 24.6; 95% CI 3.6–167.6). Twenty-eight-day ICU-free days were fewer in the NRT group.	IFE (RQ3)	NRT—formulation not specified	Pharmacological
de Jong B. et al., 2018 [8]	The Netherlands	Randomised controlled trial	Two medical-surgical ICUs in two university-affiliated teaching hospitals; mechanically ventilated adult active smokers (>10 cigarettes/day)	47	NRT vs. placebo: no difference in 30- or 90-day mortality; serious adverse events were comparable. There was more time alive without delirium, sedation, or coma by day 20, and more patients were discharged from the ICU/hospital by day 30.	IFE (RQ3)	NRT—transdermal nicotine patch	Pharmacological
Pathak V. et al., 2013 [9]	USA	Randomised controlled trial	20-bed mixed medical-surgical ICU, St Barnabas Hospital, Bronx, New York; adult smokers ≥1 pack/day, with or without mechanical ventilation	40	NRT (21 mg patch) vs. placebo: numerically shorter ICU stay (4.5 vs. 7.0 days), fewer ventilator days (1.9 vs. 3.5), and fewer days receiving sedation/analgesia in the NRT group; differences were not statistically significant in this pilot study. No deaths occurred during ICU follow-up.	IFE (RQ3)	NRT—transdermal nicotine patch	Pharmacological
Kanova M. et al., 2021 [10]	Czech Republic	Randomised controlled trial	21-bed ICU; elective major surgery patients with nicotine dependence (≥10 cigarettes/day; ≤30 days since cessation), expected ICU stay >24 h; CAM-ICU assessed daily; 21 mg patch for up to 7 days or until ICU discharge	52 (26 NRT, 26 placebo)	NRT did not reduce delirium incidence and did not significantly affect length of hospitalisation, sedation, analgesia, vasopressor use, duration of APV, or ICU stay in this cohort.	IFE (RQ3)	NRT—transdermal nicotine patch	Pharmacological
Mehta S. et al., 2015 [11]	Canada/USA	Randomised controlled trial	16 North American medical and surgical ICUs; critically ill mechanically ventilated adults; delirium screened daily using ICDSC (positive ≥4)	430 total; 420 assessed for delirium	Delirium occurred in 53.8% (226/420). A history of tobacco use was more frequent among patients with delirium than among those without delirium (31.5% vs. 16.2%). The adverse clinical course reported in the study was associated with delirium; it was not directly attributed to tobacco use history. NWS was not assessed.	SRE (RQ1)		
Mardani D. et al., 2012 [12]	Iran	Randomised controlled trial	Cardiac surgery ICU (CSICU), Chamran Heart Center Hospital, Isfahan	196	The incidence of POD was 17.34%; smoking history was associated with higher odds of POD (OR 8.358; 95% CI 1.855–37.870). Longer time to extubation and longer ICU stay were associated with POD, not directly with smoking history. Dexamethasone was associated with a lower risk of POD. NWS was not assessed.	SRE (RQ1)		
Seder DB. et al., 2011 [13]	USA	Retrospective observational cohort study	18-bed neuro-ICU; admissions for subarachnoid haemorrhage	Active smokers with SAH: NRT n = 128 vs. no NRT n = 106 (total smokers included in analysis n = 234)	NRT was associated with lower 3-month mortality (multivariable OR 0.12; 95% CI 0.04–0.37); no differences were observed in delayed cerebral ischaemia or vasospasm. Delirium and seizures were more common in the NRT group (delirium 19% vs. 9%; seizures 9% vs. 2%). The study evaluated NRT-associated outcomes among active smokers with SAH and did not assess the occurrence or clinical phenotype of NWS.	IFE (RQ3)	NRT—transdermal nicotine patch	Pharmacological
de Almeida TML et al., 2016 [19]	Brazil	Prospective observational cohort study	17-bed general ICU, university hospital	113	Agitation within the first 7 ICU days occurred in 31.8% of patients. In multivariable analysis, smoking was an independent risk factor for agitation (OR 4.49; 95% CI 1.33–15.17), alongside delirium, moderate/severe pain, and mechanical ventilation. Agitation was associated with fewer mechanical ventilation-free days at day 7.	SRE (RQ1)		
Van Rompaey B., et al., 2009 [20]	Belgium	Prospective observational cohort study	ICU; delirium assessed using the NEECHAM Confusion Scale	523 patients	Heavy smoking (≥10 cigarettes/day) was associated with an increased risk of delirium (reported OR ≈ 2.04).	SRE (RQ1)		
Cartin-Ceba R et al., 2011 [21]	USA	Prospective observational cohort study	24-bed medical ICU	330 total; NRT group n = 174 vs. no-NRT group n = 156	Hospital mortality did not differ significantly between groups. After adjustment for illness severity and the propensity to receive NRT, NRT was not associated with increased hospital mortality (OR 1.4; 95% CI 0.5–3.9). Secondary outcomes included delirium-related measures and restraint use. The study evaluated NRT-associated outcomes and did not assess the occurrence or clinical phenotype of NWS.	IFE (RQ3)	NRT—transdermal nicotine patch	Pharmacological
Avrami S et al., 2012 [22]	Greece	Prospective observational cohort study	ICU; delirium assessed with CAM-ICU; sedation assessed with RASS	122 ICU patients (stay > 48 h)	Delirium frequency was 43%; a history of smoking was associated with delirium (*p* = 0.023). Delirium was associated with increased mortality (*p* = 0.001).	SRE (RQ1)		
Jayaswal AK. et al., 2019 [23]	India	Prospective observational cohort study	ICU (tertiary care teaching hospital); delirium assessed with CAM-ICU, motor subtype with RASS	280 patients; delirium in 88 (31.4%)	Tobacco use was a significant predisposing factor for ICU delirium (*p* = 0.016). Delirium was associated with longer ICU stay and higher 1-month post-discharge mortality.	SRE (RQ1)		
Yang L. et al., 2017 [24]	China	Prospective observational cohort study	Medical and surgical ICU; mechanically ventilated patients receiving sequential sedation; delirium assessed every 4 h with CAM-ICU	141 patients	Regular smoking independently increased the risk of delirium (RR 2.366; 95% CI 1.277–4.382; *p* = 0.006). Sequential sedation with dexmedetomidine was associated with a lower risk of delirium (RR 0.448; *p* = 0.040).	SRE (RQ1)		
Huang HW et al., 2021 [25]	China	Prospective observational cohort study	Neurosurgical ICU after elective craniotomy; adults ≥ 18 years; POD assessed with CAM-ICU twice daily on postoperative days 1–3	800	Smoking history was independently associated with POD (aOR 2.582; 95% CI 1.611–4.140) within a multivariable prediction model.	SRE (RQ1)		
Dubois MJ et al., 2001 [26]	Canada	Prospective observational cohort study	16-bed medical-surgical ICU	198 with complete comparative data (38 delirium; 160 no delirium); total cohort also reported as 216 in outcome analyses	In multivariable analysis, smoking was associated with an increased risk of delirium (OR 2.2; 95% CI 0.94–4.90; borderline significance). The delirium group had higher rates of device/catheter removal (~20%) and self-extubation (~10%), with a trend towards longer ICU stay.	SRE (RQ1)		
Kerber K. et al., 2020 [27]	USA	Retrospective cohort study	Medical ICU (MICU); adult patients ≥ 18 years requiring endotracheal intubation and mechanical ventilation > 24 h	171	Hypoactive delirium was common (up to 44% within the first 7 days). Delirium was not a predictor of unplanned extubation; however, smoking history predicted unplanned extubation (OR ≈ 3.2), together with COPD (OR ≈ 5.2) and failed spontaneous breathing trials (OR ≈ 12.6).	SRE (RQ1)		
Yang M. et al., 2025 [28]	USA	Retrospective cohort study	Adult ICU stays ≥24 h with delirium assessments (CAM-ICU); nicotine dependence defined using ICD-9/ICD-10 codes; MIMIC-IV v3.1	24,043 (ND = 2662; after PSM: 2653 per group)	Nicotine dependence was associated with a higher incidence of ICU delirium (after PSM: 30.8% vs. 27.2%, *p* = 0.004) and with a higher delirium risk in multivariable models; no significant differences in mortality or length of stay were observed after matching.	SRE (RQ1)		
Tian H. et al., 2020 [29]	China	Retrospective cohort study	Tertiary hospital ICU; patients with AECOPD requiring invasive mechanical ventilation; delirium monitored with CAM-ICU	620	ICU delirium occurred in 15.0% (93/620). In multivariable analysis, current smoking was identified as a significant risk factor for ICU delirium, together with age, male sex, alcoholism with active abstinence, stage 3 AKI, and ASA III.	SRE (RQ1)		
Wang R. et al., 2025 [30]	USA	Retrospective observational cohort study	Adult ICU patients with sepsis (MIMIC-IV, 2008–2022)	10,855	Smoking was associated with a higher incidence of delirium (34.8% vs. 25.7%) and remained independently associated after adjustment (OR ~1.44; 95% CI 1.28–1.61); the association was confirmed using PSM and IPTW. PaCO_2_ partially mediated the effect (~7.9%).	SRE (RQ1)		
Zhao Y. et al., 2024 [31]	China	Retrospective observational cohort study	Post-cardiac surgery ICU (CABG patients; ≥24 h observation)	320	The incidence of POD was 29.06%. Univariate differences included smoking; multivariable risk factors were longer surgery duration, longer ICU stay, longer duration of mechanical ventilation, and higher pain scores (VAS).	SRE (RQ1)		
Kotfis K. et al., 2018 [32]	Poland	Retrospective observational cohort study	Tertiary hospital cardiac ICU after isolated CABG (postoperative intubated/mechanically ventilated patients)	1904	Intubation time >12 h increased the risk of postoperative delirium (OR 1.548) and haemofiltration; smokers were more common among those extubated earlier, with smoking measured as a baseline factor.	SRE (RQ1)		
Wu NN et al., 2023 [33]	China	Systematic review/meta-analysis	Intensive care unit settings (different countries and ICU types in the primary studies)	51 studies, 39,076 patients	Smoking was significantly associated with delirium: OR 1.55 (95% CI 1.11–2.00), 4 studies; I^2^ = 74.1%; *p* < 0.001.	SRE (RQ1)		
Ng K. T. et al., 2017 [34]	UK/Malaysia	Systematic review/meta-analysis	ICU settings (mixed medical/surgical ICU, medical ICU, neurosurgical ICU, postoperative CABG across included studies)	8 studies; total n = 2636	Meta-analysis of observational studies showed that NRT was associated with increased delirium (3 studies; n = 908; OR 4.03, 95% CI 2.64–6.15; I^2^ = 0%). No statistically significant difference in ICU mortality was found in the pooled analysis (reported OR 0.58; 95% CI 0.31–1.10). The authors concluded that routine NRT cannot currently be recommended to prevent delirium or reduce mortality because of the lack of high-quality data.	IFE (RQ3)	NRT—transdermal nicotine patch	Pharmacological
S. Jean Hsieh sj. et al., 2013 [35]	USA	Systematic review/meta-analysis	Adult ICU and surgical hospital populations (included cohort studies)	14 cohort studies (total 4382 patients)	Evidence was inconclusive and insufficient to determine whether active smoking is a risk factor for delirium; major limitations included heterogeneity and poor/variable assessment of smoking exposure, without biomarker validation.	SRE (RQ1)		
Aljuhani O. et al., 2024 [36]	Saudi Arabia	Systematic review/meta-analysis	ICU smokers randomised to receive NRT vs. no NRT/placebo during ICU stay; MEDLINE/Embase searched through 13 February 2023	3 RCTs; 139 total (67 NRT, 72 control)	Pooled results showed that NRT was associated with a shorter ICU length of stay (MD ≈ −3.06 days); no significant pooled differences were found for duration of mechanical ventilation, duration of delirium, or vasopressor duration.	IFE (RQ3)	NRT—formulation not specified	Pharmacological
Huai J. et al., 2014 [37]	China	Systematic review/meta-analysis	Critical care/ICU settings; MEDLINE and Embase search through January 2014; included studies used validated delirium tools (e.g., CAM-ICU/ICDSC/NEECHAM/Nu-DESC)	25 studies; total N = 8553	Pooled estimates indicated that smoking was not significantly associated with delirium overall (univariate OR ≈ 1.01 [0.81–1.25]; multivariate OR ≈ 1.61 [0.83–3.10]); delirium risk was associated with age, hypertension, mechanical ventilation, and higher APACHE II scores.	SRE (RQ1)		
Kebapçı A. et al., 2025 [38]	Türkiye	Retrospective case–control study	Medical-surgical ICU; intubated patients with COVID-19 (mechanical ventilation ≥48 h)	138 (prone n = 59; supine n = 79)	In the prone-positioned subgroup, smoking increased the risk of VAP (OR 8.146; 95% CI 1.297–51.161; *p* = 0.025).	SRE (RQ1)		
Komninou MA et al., 2024 [39]	Switzerland	Retrospective case–control study	Postoperative ICU patients; smoking status categorised as non-smoker/active smoker/former smoker; delirium compared across groups with matched analysis	495 total; matched analysis reported in pairs	Former smoking was associated with higher odds of delirium (unmatched OR 1.82; matched OR 3.0); active smoking did not differ significantly from non-smokers. Regression analysis confirmed former smoking as an independent factor (OR ~2.87).	SRE (RQ1)		
Locihová H. et al., 2022 [40]	Czech Republic	Prospective observational study	ICU, non-intubated patients, stay > 24 h	126 consecutive patients	Smoking was significantly associated with delirium in Kendall’s tau analyses: CAM-ICU tau = 0.191 (*p* < 0.001); ICDSC tau = 0.224 (*p* < 0.001).	SRE (RQ1)		
Tiwari AM et al., 2023 [41]	India	Prospective observational study	Adult ICU (48-bed level 3 semi-closed ICU); delirium assessed with CAM-ICU + RASS; confirmation by psychiatrist/neurophysician	936 patients included; delirium confirmed in 207 (22.11%)	Smoking history was significantly associated with delirium (Table 2; *p* < 0.05). Delirium was associated with complications such as catheter/tube removal, aspiration, reintubation, and pressure ulcers, as well as with higher mortality (21.3% vs. 5%).	SRE (RQ1)		
Vyveganathan L. et al., 2019 [42]	Malaysia	Prospective cross-sectional observational study	General ICU, teaching hospital in Kuala Lumpur; adults ≥ 18 years; ICU stay > 24 h	139	Delirium incidence was 42% (hypoactive 68%); smoking was among the significant predisposing risk factors. Delirium was associated with longer duration of mechanical ventilation and longer ICU stay.	SRE (RQ1)		
Spiropoulou E. et al., 2022 [43]	Greece	Prospective observational study	Cardiac surgery ICU, Onassis Cardiac Surgery Center	86	The incidence of POD assessed with CAM-ICU was 25.6%; smoking history was associated with POD in univariable analysis (OR 3.8; 95% CI 1.15–12.5). Other associated factors included alcohol use, COPD, ICU arrhythmia, and post-extubation hypoxaemia; multivariable predictors included post-extubation hypoxaemia, heart rate after extubation, and alcohol use.	SRE (RQ1)		
Dehghani-Ghorbi M. et al., 2025 [44]	Iran	Retrospective observational study	ICU admissions of patients with underlying cancer	90	Delirium was more frequent in patients with a smoking history (Table 2: smoking history yes, 66.7% with delirium vs. 40.5% without; *p* = 0.01). Pressure ulcers were also more frequent in patients with a smoking history (lee: smoking history yes, 81% with pressure ulcers vs. 46.4% without; *p* = 0.005).	SRE (RQ1)		
Li HR et al., 2024 [45]	China	Retrospective observational study	Emergency ICU	301	Independent risk factors for delirium included smoking history (OR 2.787). Combined emotional and pain nursing was associated with a lower risk of delirium (OR 0.351).	SRE (RQ1)		
Cai M. et al., 2025 [47]	China	Prospective observational cohort study	Single tertiary hospital (Fujian Medical University Union Hospital); postoperative cardiac surgery ICU with mechanical ventilation	216 (final analysed sample)	Smoking history was independently associated with prolonged mechanical ventilation (OR 7.417; 95% CI 2.425–22.684). Poor sleep quality (OR 11.59; 95% CI 3.844–34.942) and postoperative delirium (OR 5.10; 95% CI 1.793–14.504) were also independently associated with prolonged mechanical ventilation. NWS was not assessed.	SRE (RQ1)		
Carle CM. et al., 2012 [48]	USA	Prospective observational study; Doctoral dissertation	ICU (19-bed unit); measures collected within 24 h of admission and repeatedly during ICU stay; delirium measured with CAM-ICU, agitation with RASS, anxiety with the Faces Anxiety Scale, craving with a VAS; cotinine/cortisol also included	8 enrolled (from 105 screened; recruitment details reported)	This doctoral dissertation reported a small prospective observational pilot study describing the trajectory of nicotine withdrawal-related symptoms in critically ill smokers, including anxiety, agitation, craving, and delirium-related measures; NRT exposure was rare and at physician discretion, reflecting feasibility limitations.	WFE (RQ2); IFE (RQ3)	NRT—formulation not specified	Pharmacological
Atkins PM, et al., 1997 [49]	USA	Retrospective case–control study	Adult ICUs, tertiary referral centre	150 (50 self-extubation; 100 controls)	Cases of self-extubation were more likely to have a current smoking history (*p* < 0.05). Independent factors for self-extubation were restlessness/agitation and hospital-acquired adverse events. Self-extubation was associated with longer ICU/hospital stays and higher rates of reintubation and complications.	SRE (RQ1)		
Awissi DK et al., 2013 [50]	Canada	Narrative review	ICU	Not applicable (review)	This narrative review addressed the clinical recognition and management of alcohol, nicotine, and iatrogenic withdrawal in the ICU. For nicotine withdrawal, it discussed possible manifestations and NRT while emphasising the limited ICU-specific evidence. It did not provide primary prevalence data or a validated ICU-specific diagnostic approach for NWS.	WFE (RQ2); IFE (RQ3)	NRT—transdermal nicotine patch	Pharmacological
Honisett TD., 2001 [51]	UK	Narrative review	ICU context (review)	Not applicable	This review discussed nicotine withdrawal in smokers hospitalised in the ICU, including symptom profile and clinical recognition, and outlined nicotine replacement options, focusing on patches, as a supportive strategy to reduce withdrawal-related agitation/restlessness and potentially improve ICU tolerance. Evidence gaps and research questions were highlighted.	WFE (RQ2); IFE (RQ3)	NRT—transdermal nicotine patch	Pharmacological
Mayer SA et al., 2001 [52]	USA	Case report	Neurological ICU (neuro-ICU)	5	Five heavy smokers with brain injury developed agitated delirium 2–10 days after smoking cessation; each showed rapid clinical improvement within hours of receiving a 21 mg transdermal nicotine patch. The authors explicitly noted that causality was not proven and that the findings might reflect coincidence or underlying neurological disease, warranting confirmation in randomised trials.	WFE (RQ2); IFE (RQ3)	NRT—transdermal nicotine patch	Pharmacological
Panos NG. et al., 2010 [53]	USA	Retrospective cohort study	Neurosurgery ICU, University of Illinois Medical Center at Chicago; adults > 18 years admitted with neurological insults; groups: smokers + NRT, smokers without NRT, non-smokers	340	No difference in unfavourable discharge disposition was found between groups (*p* = 0.17). The NRT group had longer ICU and hospital length of stay; other secondary outcomes (mortality, rebleeding, ischaemic stroke) did not differ.	IFE (RQ3)	NRT—transdermal nicotine patch	Pharmacological

Evidence-focus codes: SRE, smoking-related exposure, defined as smoking history, current or former smoking, or coded nicotine dependence analysed as an exposure or associated factor; WFE, withdrawal-focused evidence, defined as abrupt nicotine cessation, NWS, or candidate withdrawal-related symptoms directly assessed, or the clinical recognition of NWS explicitly discussed; IFE, intervention-focused evidence, in which an intervention intended to prevent or alleviate nicotine withdrawal was evaluated or described. More than one code could be assigned to a source. NRT-focused sources were not classified as WFE solely because NRT was administered or evaluated to prevent withdrawal. Classification as WFE identifies the focus of the source and does not indicate that NWS was confirmed using a validated ICU-specific diagnostic instrument or that nonspecific ICU manifestations were causally attributable to withdrawal. Distribution: SRE, n = 29; WFE, n = 5; IFE, n = 15; categories were not mutually exclusive. Abbreviations: AECOPD, acute exacerbation of chronic obstructive pulmonary disease; AKI, acute kidney injury; aOR, adjusted odds ratio; APACHE, Acute Physiology and Chronic Health Evaluation; APV, artificial pulmonary ventilation; ASA III, American Society of Anesthesiologists Physical Status III; CABG, coronary artery bypass grafting; CAM-ICU, Confusion Assessment Method for the ICU; CI, confidence interval; COPD, chronic obstructive pulmonary disease; COVID-19, coronavirus disease 2019; CSICU, cardiac surgery intensive care unit; ICD-9/ICD-10, International Classification of Diseases, Ninth/Tenth Revision; ICDSC, Intensive Care Delirium Screening Checklist; ICU, intensive care unit; IFE, intervention-focused evidence; I^2^, heterogeneity statistic; IPTW, inverse probability of treatment weighting; MD, mean difference; MICU, medical intensive care unit; MIMIC-IV, Medical Information Mart for Intensive Care IV; ND, nicotine dependence; NEECHAM, Neelon and Champagne Confusion Scale; NRT, nicotine replacement therapy; Nu-DESC, Nursing Delirium Screening Scale; NWS, nicotine withdrawal syndrome; OR, odds ratio; PaCO_2_, arterial carbon dioxide tension; POD, postoperative delirium; PSM, propensity score matching; RASS, Richmond Agitation-Sedation Scale; RCT, randomised controlled trial; RR, risk ratio; RQ, review question; SAH, subarachnoid haemorrhage; SRE, smoking-related exposure; VAP, ventilator-associated pneumonia; VAS, visual analogue scale; WFE, withdrawal-focused evidence.

## Data Availability

No new data were created or analysed in this study.

## Supplementary Materials

The following supporting information can be downloaded at: https://www.mdpi.com/article/10.3390/medsci14030374/s1.
