# Supplementary material for: Nicotine Withdrawal Syndrome in Intensive Care Patients—Preventive and Therapeutic Implications
Source: Med Sci (Basel). 2026 Jul 4;14(3):374. doi: 10.3390/medsci14030374 (PMC13414422; doi:10.3390/medsci14030374)
Supplement: Supplementary file 1 [file medsci-14-00374-s001.zip › medsci-4401674-supplementary.pdf]

Search strategies:

**Keywords**	“Smoker”, “Smoking”, “Tobacco Smoking”, “Tobacco”, “Nicotine”, “Cigarette”, “Critically ill”, “Intensive Care Unit”, “Critical Care”, “ICU”, “Intensive Care”, “Critical Illness”, “Delirium”, “Agitation”, “acute agitation”, “Aggression”, “Psychomotor Agitation”, “withdrawal symptoms”
**Search strategy**	**PubMed:** (((Smoker OR Smoking OR 'Tobacco Smoking' OR Tobacco OR Nicotine OR Cigarette) AND ('Critically ill'[Title] OR 'Intensive Care Unit'[Title] OR 'Critical Care'[Title] OR ICU[Title] OR 'Intensive Care'[Title] OR 'Critical Illness'[Title])) AND (Delirium OR Agitation OR 'acute agitation' OR Aggression OR 'Psychomotor Agitation' OR 'withdrawal symptoms'[MeSH Terms])) **Limit:** Language **Results:** 49
	**CINAHL:** TX (Smoker OR Smoking OR 'Tobacco Smoking' OR Tobacco OR Nicotine OR Cigarette) AND TI ('Critically ill' OR 'Intensive Care Unit' OR 'Critical Care' OR ICU OR 'Intensive Care' OR 'Critical Illness') AND TX (Delirium OR Agitation OR 'acute agitation' OR Aggression OR 'Psychomotor Agitation' OR 'withdrawal symptoms') **Limit:** Language **Results:** 338
	**Web of Science:** ((TS=(Smoker OR Smoking OR 'Tobacco Smoking' OR Tobacco OR Nicotine OR Cigarette)) AND TS=('Critically ill' OR 'Intensive Care Unit' OR 'Critical Care' OR ICU OR 'Intensive Care' OR 'Critical Illness')) AND TS=(Delirium OR Agitation OR 'acute agitation' OR Aggression OR 'Psychomotor Agitation' OR 'withdrawal symptoms') **Limit:** Language **Results:** 117
	**Cochrane Library:** (Smoker OR Smoking OR 'Tobacco Smoking' OR Tobacco OR Nicotine OR Cigarette) AND ('Critically ill' OR 'Intensive Care Unit' OR 'Critical Care' OR ICU OR 'Intensive Care' OR 'Critical Illness') AND (Delirium OR Agitation OR 'acute agitation' OR Aggression OR 'Psychomotor Agitation' OR 'withdrawal symptoms') **Limit:** Language **Results:** 122
	**Scopus:** (ALL (Smoker OR Smoking OR Tobacco Smoking OR Tobacco OR Nicotine OR Cigarette) AND TITLE-ABS-KEY (Critically ill OR Intensive Care Unit OR Critical Care OR ICU OR Intensive Care OR Critical Illness) AND ALL (Delirium OR Agitation OR acute agitation OR Aggression OR Psychomotor Agitation OR withdrawal symptoms)) AND (LIMIT-TO (LANGUAGE , "English")) **Limit:** Language **Results:** 28
